# Taponamiento Cardíaco por Ruptura de Aneurisma Coronario Gigante sobre Fístulas Coronarias: Reporte de un Caso Inusual

**DOI:** 10.47487/apcyccv.v1i2.25

**Published:** 2020-06-29

**Authors:** Francisco Chávez-Solsol,, Luis Murillo-Pérez, Ricardo Heredia-Oré, Zoila Rodríguez-Urteaga, María Toribio-Salazar, Yemmy Pérez-Valverde

**Affiliations:** 1 Servicio de Cardiología Clínica. Instituto Nacional Cardiovascular - INCOR EsSalud. Lima, Perú. Servicio de Cardiología Clínica Instituto Nacional Cardiovascular - INCOR EsSalud Lima Perú; 2 Médico residente de Cardiología. Instituto Nacional Cardiovascular - INCOR EsSalud. Lima, Perú. Instituto Nacional Cardiovascular - INCOR EsSalud Lima Perú; 3 Servicio de Ayuda al Diagnóstico y Tratamiento. Instituto Nacional Cardiovascular - INCOR EsSalud. Lima, Perú. Servicio de Ayuda al Diagnóstico y Tratamiento Instituto Nacional Cardiovascular - INCOR EsSalud Lima Perú; 4 Médico residente de Cirugía de Tórax y Cardiovascular. Instituto Nacional Cardiovascular - INCOR EsSalud. Lima, Perú. Instituto Nacional Cardiovascular - INCOR EsSalud Lima Perú; 5 Servicio de Cirugía Cardiovascular Adulto. Instituto Nacional Cardiovascular - INCOR EsSalud. Lima, Perú. Servicio de Cirugía Cardiovascular Adulto Instituto Nacional Cardiovascular - INCOR EsSalud Lima Perú

**Keywords:** fístula coronaria, aneurisma coronario, taponamiento cardíaco, coronary artery fistula, coronary aneurysm, cardiac tamponade

## Abstract

Las fístulas de arterias coronarias son entidades clínicas infrecuentes y su asociación con aneurismas coronarios gigantes es aun más inusual. La mayoría de las fístulas son asintomáticas, pero los aneurismas podrían desarrollar síntomas según su diámetro. Presentamos el caso clínico de un paciente con dolor torácico y arresto cardíaco, que posteriormente desarrolló taponamiento cardíaco con necesidad de cirugía de emergencia, por ruptura de aneurisma coronario gigante desarrollado a partir de fístulas de arterias coronarias confluentes desde dos arterias coronarias hacia la arteria pulmonar.

## Abstract (en)

Coronary artery fistulas are rare clinical entities and their association with giant coronary aneurysms is even more unusual. Most fistulas are asymptomatic, but aneurysms could develop symptoms depending on their diameter. We present a patient with chest pain and cardiac arrest, who later developed cardiac tamponade that needed emergency surgery, due to rupture of a giant coronary aneurysm developed from confluent coronary artery fistulas from two coronary arteries to pulmonary artery.

## Introducción

Se define como fístula de arterias coronarias (FAC) a la comunicación anormal adquirida o congénita de una arteria coronaria con las cámaras cardíacas o con algún segmento de la circulación pulmonar o sistémica. ^(^^1^ Es una anomalía poco frecuente, con una prevalencia de hasta 0.9% en la población general.^2^ La mayoría son únicas, y sólo en 10% a 16% de casos se reportan FAC múltiples.^3^ Aproximadamente el 55% de FAC se originan de la coronaria derecha, 35% de la coronaria izquierda y sólo el 10% de ambas arterias. Asimismo, el drenaje más frecuente es hacia cavidades cardíacas derechas o hacia circulación pulmonar^4^

La mayoría de las FAC son asintomáticas, pero si el cortocircuito permite un flujo sanguíneo importante, puede generar falla cardíaca, angina o endocarditis activa. ^(^^5^ Por otro lado, alrededor del 20% de las FAC pueden acompañarse de formación de aneurismas de arterias coronarias (AAC), usualmente solitarios^4^ sin embargo, la ocurrencia de aneurismas múltiples sobre un trayecto fistuloso constituye una entidad extremadamente rara y más aún cuando éstos son gigantes (diámetro mayor de 20 mm). ^6^ Asimismo, esta asociación puede complicarse con ruptura, complicación muy poco frecuente y con una alta tasa de mortalidad, ^2^ especialmente cuando el diámetro del aneurisma es mayor a 30 mm. ^7^

## Descripción del Caso

Se presenta el caso de un paciente con fístulas de arterias coronarias hacia arteria pulmonar con formación de dos aneurismas gigantes sobre el trayecto fistuloso, complicado con arresto cardíaco, ruptura y taponamiento cardíaco.

Paciente varón de 51 años, ex-tabaquista, natural y procedente de Cusco. Ingresó a nuestra institución con tiempo de enfermedad de 3 días, de inicio brusco, caracterizado por dolor torácico punzante de intensidad 3/10, y posterior pérdida súbita de conciencia. Fue trasladado a un hospital de su localidad, donde ingresó por arresto cardíaco y se realizó reanimación cardiopulmonar exitosa. Durante la hospitalización presentó el hallazgo de aneurisma coronario y derrame pericárdico leve, por lo cual fue referido a nuestra institución.

El paciente ingresó a nuestra institución con presión arterial (PA) de 133/81 mmHg, frecuencia cardíaca (FC) en 71 latidos por minuto, frecuencia respiratoria de 19 respiraciones por minuto, saturación de oxígeno 94% a FiO_2_ 32%, en regular estado general, nutrición e hidratación. En el examen físico se encontró ruidos cardíacos rítmicos de buena intensidad, sin soplos. Además, presentó crepitantes en el tercio inferior de ambos hemitórax. El resto del examen no mostró alteraciones significativas.

**Figura 1:**
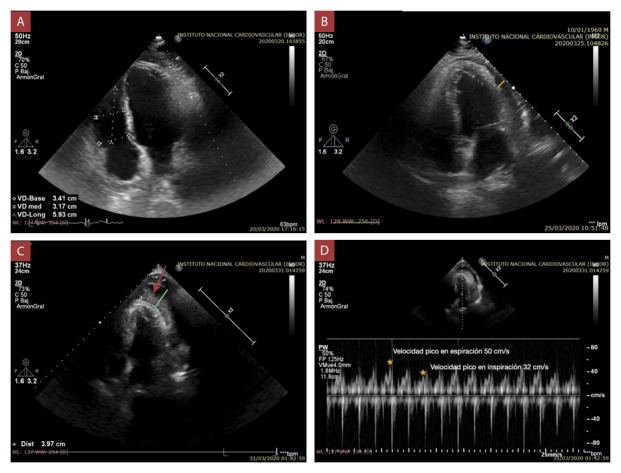
Ecocardiografía transtorácica vista apical 4 cámaras.

El electrocardiograma, la radiografía de tórax y el laboratorio de rutina no mostraron alteraciones significativas. Se realizó una ecocardiografía (Figura 1A) mostrando una fracción de eyección de ventrículo izquierdo (FEVI) de 60%, TAPSE 27 mm, cavidades cardíacas no dilatadas sin alteración de motilidad, no valvulopatías, ni derrame pericárdico.

A) Sin evidencia de derrame pericárdico al ingreso. B) 5° día hospitalario con derrame pericárdico leve de 4 mm (línea amarilla). C) 10° día hospitalario con derrame pericárdico severo de hasta 23.4 mm (línea verde) con imagen de coágulo adherido a pared ventricular izquierda (flecha roja). D) Variabilidad del flujo doppler pulsado trans-mitral mayor al 35%.

**Figura 2:**
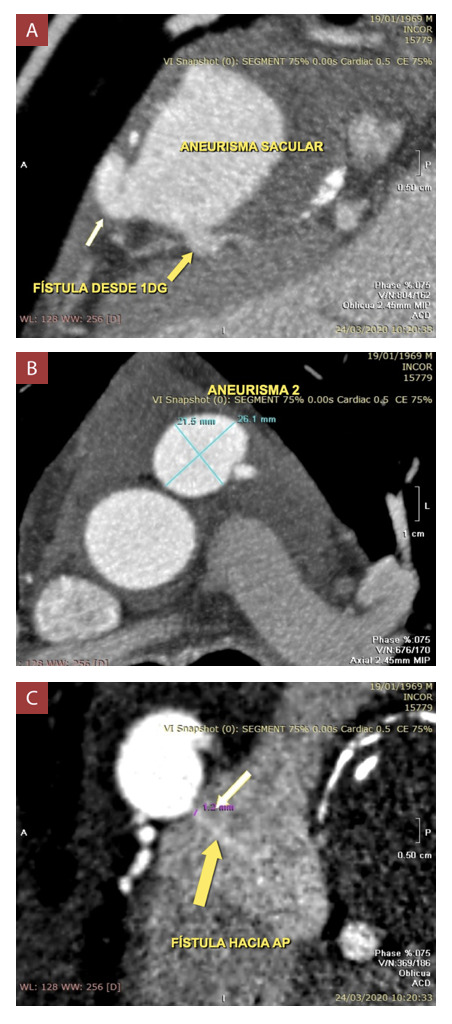
Angiotomografía coronaria.

Se realizó angiotomografía coronaria (Figura 2 y 3, Video 1) en la que se observó dos fístulas coronarias, una de ellas desde la primera rama diagonal, y la otra desde la arteria conal derecha, ambas con drenaje hacia la arteria pulmonar; además cada una presentaba un aneurisma sacular en su trayecto, el mayor de ellos dependiente de la fístula originada en la primera rama diagonal, con una dimensión de 54.5 x 45 x 45 mm, calcificado y con trombo mural. El diámetro de la comunicación de ambas fístulas con la arteria pulmonar fue de 1.2 mm, además, se encontró derrame pericárdico. La coronariografía reafirmó la presencia de aneurisma de la primera rama diagonal y arteria conal derecha, con fístula hacia arteria pulmonar. (Figura 4)

A) Aneurisma gigante (Aneurisma 1) dependiendo de fístula desde la primera rama diagonal (Fístula 1), con trombo mural y paredes calcificadas. B) Aneurisma gigante (Aneurisma 2) dependiendo de fístula desde la arteria conal derecha (fístula 2). C) Comunicación de fístulas coronarias con arteria pulmonar.

**Figura 3:**
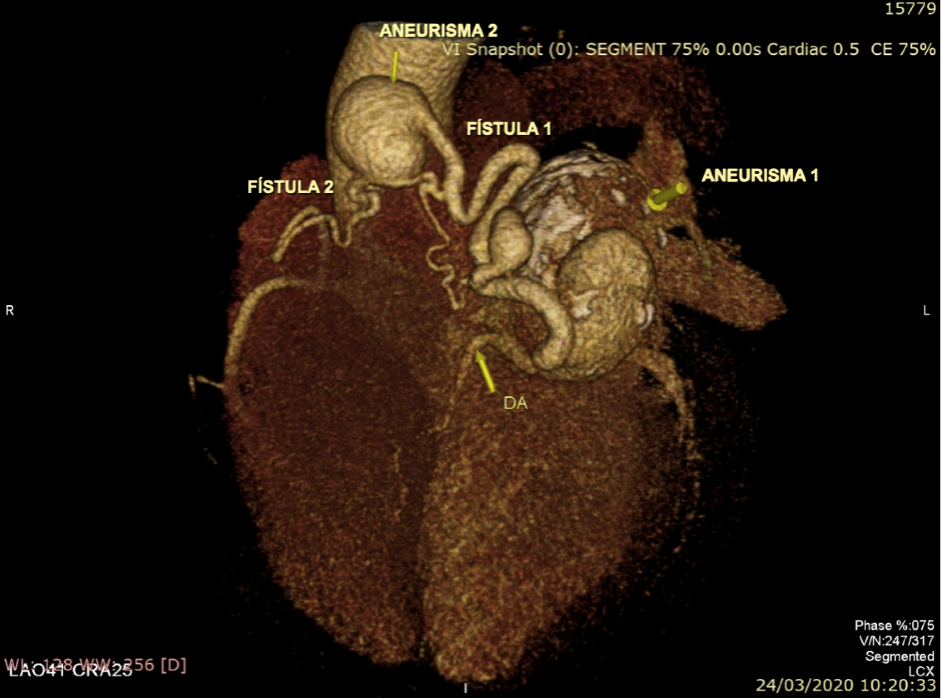
Reconstrucción tridimensional de angiotomografía coronaria que muestra las fístulas y aneurismas gigantes.

**Figura 4:**
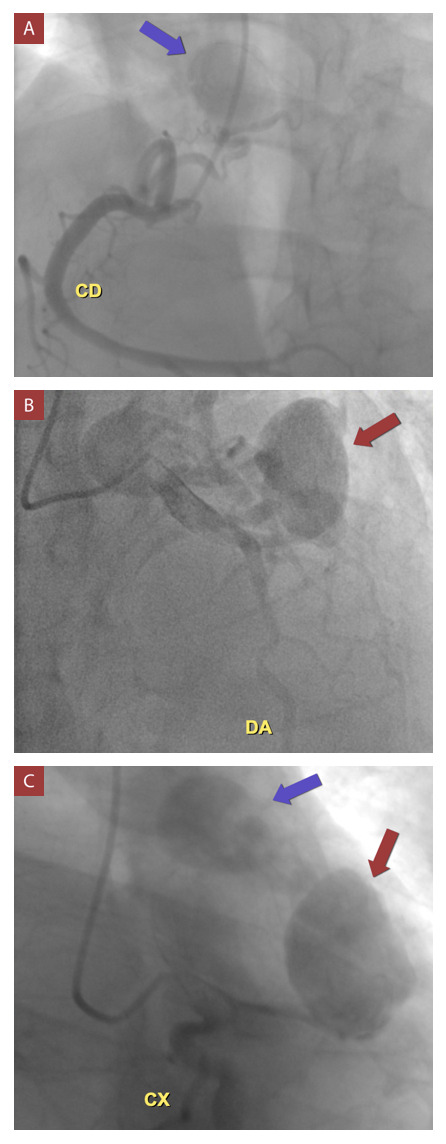
Coronariografía.

A) Aneurisma gigante 2 (flecha azul) dependiente de fístula proveniente de rama conal. B) Aneurisma gigante 1 (flecha azul) dependiente de fístula proveniente de primera rama diagonal. C) Comunicación de flujos de ambos aneurismas (flecha roja y azul) con pasaje de contraste mínimo hacia arteria pulmonar. CD: arteria coronaria derecha; DA: arteria descendente anterior; CX: arteria circunfleja.

Al quinto día de hospitalización, el control ecocardiográfico mostró derrame pericárdico leve (Figura 1B) y cursó con evolución estacionaria; sin embargo, al décimo día de hospitalización, súbitamente presentó alteración del estado de conciencia, hipotensión arterial sostenida (PA 66/33 mmHg), diaforesis, y taquipnea. La ecocardiografía (Figura 1C y 1D) evidenció derrame pericárdico severo con compromiso hemodinámico, e imagen compatible de trombo adherido a la pared del ventrículo izquierdo. Se realizó pericardiocentesis obteniendo 30 ml de contenido hemático.

**Figura 5:**
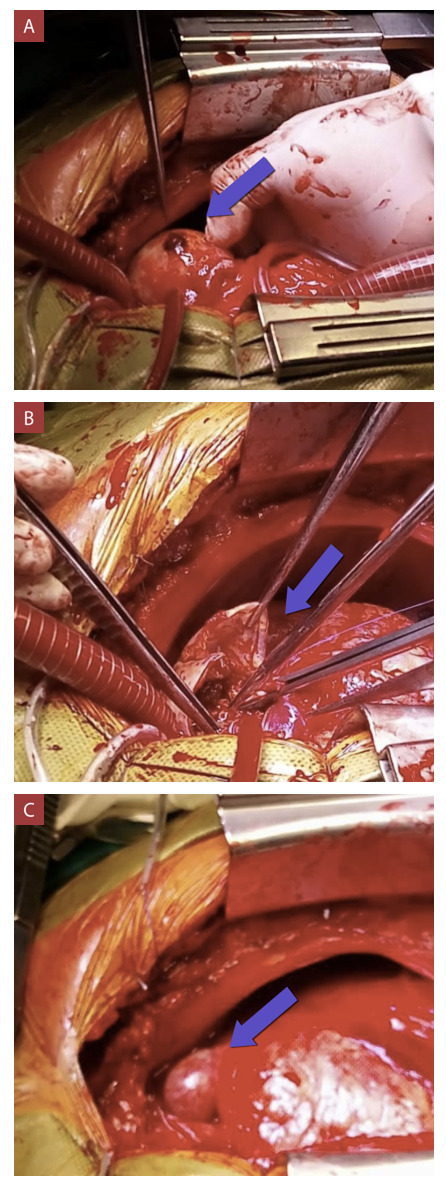
Procedimiento quirúrgico.

Ante la sospecha de ruptura de aneurisma coronario, se programó cirugía de emergencia. Luego de esternotomía media se evidenció hemopericardio de 600 ml y se ingresó a circulación extracorpórea sin parada cardíaca. Se halló un coágulo adherido a malformación vascular con un primer aneurisma sacular calcificado, roto, de 50 mm, originado de fístula coronaria dependiente de un sub-ramo de la primera diagonal; y un segundo aneurisma sacular de 30 mm, no roto, originado de fístula coronaria dependiente de arteria conal derecha. Se identificó y separó la arteria de alimentación y cada extremo se cerró con sutura de polipropileno 4/0. (Figura 5) Al abrir el aneurisma gigante roto se evidenció paredes calcificadas con detritus de contenido aterosclerótico y trombo de aproximadamente 10 mm que ocluía orificio del aneurisma, además de una fístula hacia arteria pulmonar que se excluyó. El aneurisma de 30 mm se identificó y se separó con misma técnica descrita previamente, y a su apertura se evidenció una fístula hacia arteria pulmonar que también fue excluida. Las paredes aneurismáticas se cerraron con polipropileno 4/0 (Fig. 4). Paciente no requirió puentes aorto-coronarios, y la cirugía se realizó sin intercurrencias.

A) Aneurisma 1 sacular calcificado roto de 50mm, originado de fístula dependiente de primera diagonal (flecha azul indica orificio). B) El mismo aneurisma ya abierto, con paredes calcificadas y trombo de aproximadamente 10 mm (flecha azul). C) Aneurisma 2 sacular de 30 mm originado de la fístula de la arteria conal derecha (flecha azul).

La evolución post-quirúrgica fue favorable. El paciente presentó atelectasia basal derecha como única complicación que respondió satisfactoriamente a la fisioterapia respiratoria, y fue dado de alta al séptimo día post-quirúrgico.

## Discusión

La FAC es una anomalía infrecuente, que representa el 14% de las malformaciones coronarias congénitas.^4^ La mayoría se producen durante la etapa embrionaria, pues en este periodo las cavidades cardíacas no compactadas se encuentran en comunicación con las arterias coronarias. ^8^ Su drenaje es más frecuente hacia las cavidades derechas y dentro de este grupo, la arteria pulmonar representa el 17% de casos.^4^ Más de la mitad de pacientes mayores de 20 años de edad presentarán síntomas, de acuerdo a la severidad del cortocircuito de izquierda a derecha.^9^ El flujo a través de la FAC ocurre a lo largo del ciclo cardíaco; sin embargo, a mayor longitud, más probabilidad de desviación del flujo hacia el trayecto fistuloso y consecuente disminución del flujo coronario normal, síndrome conocido como robo coronario, que se relaciona a síntomas isquémicos que pueden llevar a arresto cardíaco,^10^ manifestación clínica que presentó nuestro paciente y que podría explicarse por este fenómeno.

Las complicaciones de las FAC incluyen falla cardíaca, hipertensión pulmonar, isquemia miocárdica y arritmias.^1^ Además, se ha reportado la asociación con AAC en aproximadamente 20% de los casos. El AAC es una complicación singular que afecta en mayor medida al sexo masculino y adultos mayores. Se clasifica según su morfología (sacular o fusiforme), según la estructura de la pared del vaso (aneurisma verdadero, pseudoaneurisma, placa compleja con apariencia aneurismática y segmento normal con apariencia aneurismática), y según diámetro (aneurisma gigante: diámetro > 20 mm).^11^ Cuando se presentan de manera aislada sobre arterias coronarias normales, la coronaria derecha (CD) es la que se afecta con mayor frecuencia en alrededor del 60%, seguido de la arteria descendente anterior (DA) en 32%, y en menor frecuencia el tronco de la coronaria izquierda en 3.5% de los casos; ^12^ sin embargo, la presencia de AAC, sobretodo gigantes, son muy inusuales en las FAC.^3^ La presencia de AAC gigantes rotos asociado a una FAC ha sido reportada anteriormente; sin embargo, la dependencia de más de una FAC no se describe en reportes de casos aislados previos,^6^ lo cual hace relevante el caso presentado, ya que los dos AAC gigantes se encontraban sobre las fístulas de dos arterias coronarias distintas, la conal y la primera diagonal, ramas de la CD y DA respectivamente, con drenaje de ambas hacia la arteria pulmonar.

Los mecanismos que explican la formación de AAC sobre FAC incluyen ateroesclerosis, defectos congénitos, desórdenes inflamatorios o del tejido conectivo, infecciones, traumas y iatrogenias, que conllevan a la destrucción de la túnica media, reemplazo por tejido conectivo hialinizado y posterior dilatación aneurismática.^13^ Por otro lado, las FAC presentadas estarían en relación a una etiología congénita y la ausencia de síntomas se explicaría por un cortocircuito de izquierda a derecha no significativo. Además, con el transcurso de los años, el desarrollo de AAC gigantes asociados a estas fístulas podrían relacionarse a cambios ateroscleróticos ante la evidencia de calcificación coronaria en las imágenes tomográficas, representando el punto intermedio de esta cadena que concluiría con la posterior ruptura y taponamiento cardíaco.

El tratamiento de las FAC depende de su anatomía, los síntomas y las complicaciones asociadas. El tratamiento de las FAC sintomáticas o complicadas suele ser quirúrgico. Se ha reportado que los AAC mayores de 30 mm de diámetro tienen mayor riesgo de ruptura;^4^ sin embargo, Sakao T y colaboradores reportan el caso de una fístula complicada con un aneurisma de 10 mm de diámetro y taponamiento cardíaco.^14^ Por ello, considerando los criterios de Konno, las FAC asociadas a AAC, independientemente del tamaño, son de indicación quirúrgica. Por otro lado, el manejo de las fístulas pequeñas y asintomáticas usualmente es conservador ya que su evolución es lenta y en muchos casos ocurre el cierre espontáneo.^9^ Sin embargo, el paciente cursó con ruptura de un aneurisma gigante sobre las FAC y taponamiento cardiaco^15^ y cuyo tratamiento definitivo es quirúrgico. No obstante, la evolución del paciente fue favorable.

## Financiación:

Ninguna
